# Porcine Small Intestinal Submucosa Extracellular Matrix: A Meta-Analysis of Composition, Processing Techniques, and Biomedical Applications

**DOI:** 10.3390/ijms26136276

**Published:** 2025-06-29

**Authors:** Tamas Toth, Radu-Alexandru Prisca, Noemi Ballo, Ana-Maria Prisca, Emoke Andrea Szasz, Angela Borda

**Affiliations:** 1 Institution Organizing University Doctoral Studies (IOSUD), George Emil Palade University of Medicine, Pharmacy, Science and Technology of Târgu Mureş, 540142 Târgu Mureș, Romania; doctothtamas@gmail.com; 2 Pediatric Surgery and Orthopedics Department, Târgu Mureș Emergency Clinical County Hospital, 540136 Târgu Mureș, Romania; 3 Azareea Medicals Târgu Mureș, 540353 Târgu Mureș, Romania; 4 Lotus Life Târgu Mureș, 540084 Târgu Mureș, Romania; 5 Histology Department, George Emil Palade University of Medicine, Pharmacy, Science and Technology of Târgu Mureş, 540142 Târgu Mureș, Romania

**Keywords:** porcine SIS-ECM, extracellular matrix, biological scaffold, wound healing

## Abstract

Porcine small intestinal submucosa (SIS) extracellular matrix (ECM) has emerged as a widely researched biological scaffold in regenerative medicine. This meta-analysis examines 205 peer-reviewed studies published from 1993 to 2025, emphasizing the biochemical composition, decellularization techniques, biomedical applications, and validation methods associated with SIS-ECM. Findings reveal a dominant focus on wound healing and cardiovascular repair, reflecting the scaffold’s mechanical adaptability and bioactivity. Research has evolved from basic compositional analyses to complex, application-specific investigations that incorporate in vivo models and functional outcome measures. Decellularization methods vary significantly, trending toward hybrid, multi-step protocols that preserve ECM integrity while ensuring cellular clearance. While the results are encouraging, a notable transparency issue persists, with only a third of studies providing full methodological details, which restricts reproducibility. Frequently utilized validation methods encompass mechanical testing, histology, DNA quantification, and growth factor retention. This review emphasizes the critical requirement for standardized guidelines in preparation and reporting to guarantee the safe and effective clinical application of SIS-ECM. Through continuous refinement and collaboration among various disciplines, SIS-ECM has the potential to serve as a next-generation scaffold for a range of regenerative uses.

## 1. Introduction

The extracellular matrix (ECM) is a dynamic network of proteins, glycoproteins, proteoglycans, and signaling molecules that provides structural support to cells while also playing a role in migration, proliferation, and differentiation.

Throughout evolutionary history, ECM structures have been remarkably conserved, highlighting their essential role in maintaining tissue architecture and function [1,2]. In addition to providing mechanical stability, the ECM actively regulates cell migration, proliferation, differentiation, and the response to injury [3].

ECM has gained interest in regenerative medicine as a biomimetic scaffold that promotes tissue repair and regeneration. Among ECM-derived biomaterials, porcine small intestinal submucosa (SIS) has become one of the most studied and clinically applied.

SIS-ECM offers inherent advantages including anatomical compatibility with human tissues, relative abundance, cost-effectiveness, and preserved bioactivity [4,5].

Its native composition—dominated by type I and III collagens, elastin, glycosaminoglycans (GAGs), fibronectin, laminin, and embedded growth factors such as VEGF and b-FGF—supports cellular integration and promotes regenerative healing responses [6,7,8].

Early clinical applications of SIS date back to the 1990s, where it was first explored for hernia repair and cardiovascular patches. Since then, SIS-derived scaffolds have been incorporated into a wide range of regenerative strategies spanning wound healing, urologic reconstruction, gastrointestinal repair, and dental/maxillofacial surgeries [9,10,11].

Several SIS products have received regulatory approval, including U.S. FDA clearance for specific indications, further highlighting its translational significance. Despite this success, critical challenges remain.

Variability in decellularization methods, donor tissue heterogeneity, and batch-to-batch processing inconsistencies can affect the final scaffold’s mechanical and biochemical properties [12,13].

Incomplete decellularization risks inflammatory responses and fibrosis, whereas excessive processing may diminish essential bioactivity [14,15].

Moreover, a lack of standardized reporting across studies limits reproducibility and slows clinical translation. With growing interest in personalized regenerative therapies, novel SIS modifications such as chemical crosslinking, bioactive molecule incorporation, and stem cell seeding are being explored to enhance scaffold functionality [16].

Given the rapidly expanding but heterogeneous body of research, a systematic meta-analysis is critical to synthesize existing knowledge, identify trends, and highlight key areas for future innovation.

This study aims to evaluate the biochemical composition, decellularization strategies, biomedical applications, and immunological considerations associated with porcine SIS-ECM, thereby offering a consolidated reference for researchers, clinicians, and biomaterial developers.

## 2. Results

**Figure 1:**
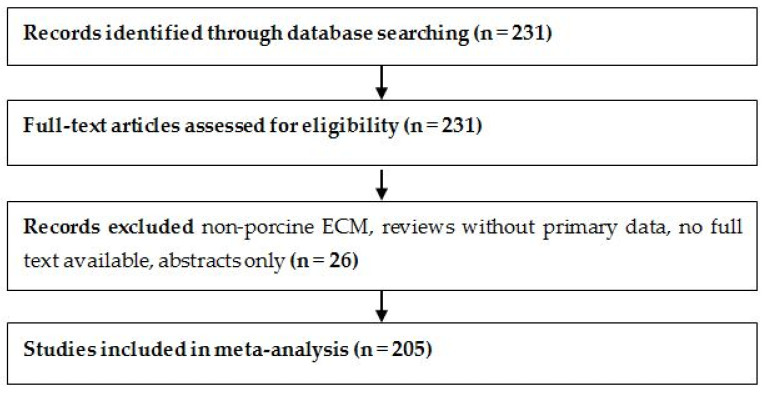
PRISMA flow diagram illustrates the selection of 205 studies included in the SIS-ECM meta-analysis.

The study selection process is summarized in Figure 1, following PRISMA 2020 guidelines [17]. A total of 231 full-text articles were assessed for eligibility. After excluding 26 studies that focused on non-porcine ECM sources, lacked full-text access, or did not report original experimental data, 205 studies were included in the final meta-analysis.

**Figure 2:**
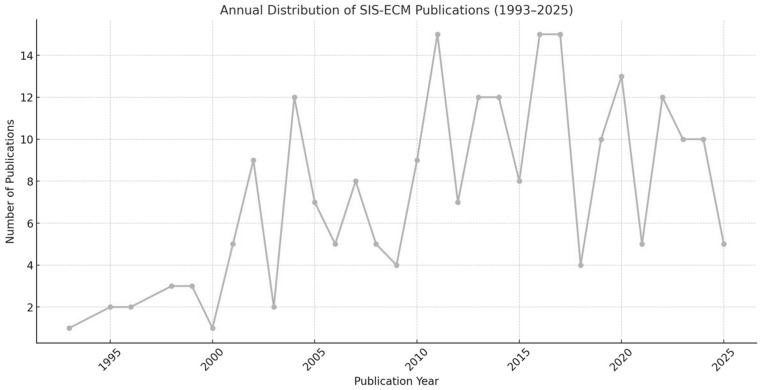
Annual distribution of SIS-ECM publications (1993–2025), based on the 205 included studies.

These studies, conducted between 1993 and 2025, show a clear upward trend in publication volume, particularly after 2010. Of the 205 studies analyzed, 156 (76.1%) were published between 2011 and 2025, confirming the growing research interest in SIS-ECM over the past decade and a half. This trend is illustrated in Figure 2, which shows the annual distribution of SIS-ECM-related publications.

Most of these studies came from biomedical and surgical research groups, with contributions from North America, Europe, and East Asia.

Regarding the applications across biomedical fields summarized in Table 1, porcine SIS-ECM was most commonly used in wound healing and soft tissue repair (34%), followed by cardiovascular reconstruction (22%), urologic applications (15%), gastrointestinal surgery (12%), and dental/maxillofacial regeneration (8%). Less common applications included orthopedic scaffolds, neural repair, and ocular surface reconstruction.

The distribution of biomedical applications for SIS-ECM highlights a strong emphasis on tissue types where regeneration is critical, with wound healing (34%) and cardiovascular reconstruction (22%) leading the field. This trend highlights the specific advantages of SIS-ECM in these contexts, particularly its high biocompatibility, angiogenic support, and ability to integrate with native tissue matrices. Growing interest in urologic (15%) and gastrointestinal (12%) applications suggests expanding clinical utility, especially in areas where soft tissue repair or functional continuity is essential. The “Other” category (9%) includes emerging applications such as neural regeneration, ocular repair, and ligament reconstruction—fields that, although currently limited in study volume, may become future focal points in SIS-ECM research.

To better understand the preparation process, we examined decellularization methods across studies.

Regarding the decellularization methodologies used, as summarized in Table 2, 84 out of 205 studies explicitly detailed their decellularization protocols. Of these, 41 utilized detergent-based chemical decellularization, most commonly SDS (sodium dodecyl sulfate), Triton X-100, or CHAPS. Twenty-nine studies employed enzymatic agents (trypsin or DNase/RNase), while 14 used physical techniques such as freeze–thaw cycles, pressure gradients, or agitation-based protocols. An increasing number of studies (post-2015) reported hybrid or multi-step protocols to preserve ECM structure and bioactivity.

Table 2 highlights the diversity of techniques used for SIS-ECM decellularization, a critical factor in preserving the biological structure and functionality of the scaffold. Chemical protocols, particularly those using detergents like SDS and Triton X-100, remain the most frequently employed (41 studies) due to their effectiveness in removing cellular material. However, reliance on harsh agents can negatively impact the bioactive composition of the ECM. Enzymatic treatments, such as DNase and trypsin (29 studies), provide a milder option, while physical methods (14 studies) effectively remove cells with limited impact on structure. A significant trend is the increasing adoption of hybrid protocols (23 studies) that blend chemical, enzymatic, and physical strategies to harmonize effectiveness with ECM preservation. Almost half of the analyzed studies (98) failed to clearly specify their decellularization method, highlighting a significant gap in methodological transparency.

Regarding trends in experimental design and validation summarized in Table 3, the analysis revealed a clear evolution in the sophistication of study designs. While early studies emphasized ECM composition and mechanical testing in vitro, more recent works (post-2015) incorporated in vivo models, functional performance metrics, and immunogenicity assessments. Among the 205 included studies

- 132 included mechanical testing;
- 98 reported histological staining and imaging;
- 74 measured DNA content or decellularization efficacy;
- 61 examined growth factor retention;
- 45 assessed host immune response or inflammatory markers.

**Table 3:** Common validation techniques in SIS-ECM studies.

Validation Method	Number of Studies
Mechanical Testing	132
Histological Analysis	98
DNA Quantification	74
Growth Factor Assays	61
Immunogenicity Evaluation	45

Beyond preparation, methodological rigor in scaffold validation was also assessed.

In terms of accessibility and transparency, only 68 studies (33%) provided fully accessible data, including detailed protocols, validation metrics, and reproducibility indicators. The remaining studies were either limited to summary metrics or lacked clarity on the efficacy of decellularization and scaffold validation. This gap underscores the need for standardized reporting guidelines across ECM studies.

The analysis of validation strategies used in the included studies (Table 3) reflects the scientific maturation of the field. Mechanical testing (132 studies) remains the most widely applied evaluation method, essential for determining the structural robustness of the scaffold under varying mechanical loads. Histological analysis (98 studies) and DNA quantification (74 studies) provide critical insight into preserving decellularization efficacy and ECM architecture. Growth factor retention assays (61 studies) indicate growing interest in maintaining scaffold bioactivity, while immunogenicity assessments (45 studies) have gained prominence in response to clinical demands for long-term safety. The variety of validation methods demonstrates a shift towards greater scientific rigor; however, it also indicates an inconsistent application of techniques among studies, reinforcing the necessity for cohesive, standardized evaluation protocols.

## 3. Discussion 

The findings of this meta-analysis reaffirm the growing body of evidence supporting the clinical and translational potential of porcine small intestinal submucosa (SIS) extracellular matrix (ECM) in regenerative medicine [3,4,5,6]. A key advantage of SIS-ECM lies in its biochemical composition, particularly its retention of structural proteins (collagen types I and III), glycosaminoglycans, and growth factors such as VEGF and b-FGF [4,5,7]. These elements are consistently associated with enhanced tissue remodeling, neovascularization, and favorable host integration across various applications, including wound healing, urologic repair, and gastrointestinal reconstruction [8,9,10,11].

Over the past three decades, research methodology related to SIS-ECM has undergone notable changes. Initially, studies concentrated on in vitro analyses, focusing on the ECM’s structural and histological features and its basic compatibility with host cells. In contrast, recent research shows a clear pivot towards more intricate experimental frameworks that include in vivo animal models, functional outcome assessments, and translational aims. These advanced designs better mirror the actual therapeutic settings, facilitating a comprehensive evaluation of regenerative capacity, immunomodulation, and mechanical performance in physiological conditions.

The approach to SIS-ECM decellularization has also undergone meaningful refinement. Initially, harsh chemical agents such as sodium dodecyl sulfate (SDS) or Triton X-100 were used extensively to achieve cellular clearance. While effective, these protocols frequently compromised the structural and bioactive components of the ECM. Contemporary strategies now favor multi-step processes that integrate enzymatic treatments, mild detergents, and physical methods (e.g., freeze–thaw cycles), emphasizing preserving ECM integrity and function. This methodological shift is reflected in more sophisticated validation practices, which increasingly rely on quantifiable metrics such as DNA content, mechanical testing, and the retention of bioactive proteins. As shown in Table 3, 132 studies included mechanical testing, while only 45 evaluated immunogenicity, highlighting the need for a more balanced scaffold evaluation.

Despite advancements, challenges remain. Commercial SIS-ECM products exhibit variability in decellularization effectiveness, sometimes leading to residual immunogenic materials or inconsistent mechanical properties. Such inconsistencies are especially crucial in high-stress contexts, like vascular or valvular repair, where instances of long-term graft failure or calcification have been documented. These performance issues underscore the broader challenge of immune compatibility in xenogeneic scaffolds. The potential presence of α-gal epitopes in certain SIS materials continues to raise worries regarding chronic inflammation and immune rejection in human recipients.

The increasing complexity of SIS-ECM research underscores the need for greater methodological standardization. Establishing unified protocols for scaffold preparation, decellularization validation, and preclinical testing will reduce inter-study variability and improve clinical predictability. Setting such benchmarks will also accelerate regulatory approval and facilitate the integration of SIS-ECM products into routine clinical workflows. Additionally, attention must be given to donor variability, batch-to-batch reproducibility, and the potential for synergistic use with stem cells, growth factors, or synthetic biomaterials.

Despite the extensive data analyzed, this meta-analysis has several limitations. Variability in reporting standards, incomplete methodological details in nearly two-thirds of the studies, and the lack of quantitative meta-analytic synthesis due to heterogeneity limit the strength of the conclusions. Additionally, publication bias and underreporting of negative results may distort the perceived efficacy of SIS-ECM applications.

In summary, while SIS-ECM continues to show promise as a biologically active scaffold for tissue repair, its future utility depends on the rigorous optimization and standardization of processing methods. With sustained research and refinement, SIS-ECM could evolve into a reliable and widely deployable component of next-generation regenerative therapies.

In addition to scientific challenges, economic and regulatory considerations also influence the clinical uptake of SIS-ECM. The high cost of advanced decellularization equipment, variability in tissue sourcing, and the need for lot-to-lot consistency impose manufacturing and compliance hurdles. Regulatory frameworks, including FDA and EMA guidelines, require rigorous documentation of scaffold preparation, sterilization, and biocompatibility testing, which not all studies address systematically. Aligning academic protocols with regulatory expectations will be crucial for the broader market adoption of SIS-based products.

Another underexplored area is the species-specific response to xenogeneic ECMs. While porcine SIS shares structural similarities with human tissue, immunological differences remain a barrier, particularly in patients with heightened sensitivity to xenogeneic proteins or α-gal epitopes. Most studies rely on rodent or porcine in vivo models, which do not fully recapitulate human immune responses. Bridging this translational gap requires expanded large-animal studies and longer-term clinical trials to assess durability and immune tolerance.

## 4. Materials and Methods

A comprehensive and systematic search of the literature identified relevant studies examining the porcine small intestinal submucosa (SIS) extracellular matrix (ECM), focusing on its biochemical composition, decellularization techniques, biomedical applications, and immunological responses. The search strategy was implemented across three major academic databases: PubMed, MDPI, and ScienceDirect, targeting articles published between 1993 and 2025. The search terms included combinations of the following keywords: “porcine” OR “pig”, “small intestinal submucosa” OR “SIS”, “extracellular matrix” OR “ECM”, and “decellularization”, “biocompatibility”, “wound healing”, or “tissue engineering”. Boolean operators were employed to enhance the specificity and breadth of the search.

To ensure data quality, the search was limited to peer-reviewed articles in full text written in English. Studies were included if they specifically examined porcine SIS-ECM and reported original experimental or clinical data related to ECM composition, decellularization protocols, immunogenic responses, or therapeutic outcomes. Studies were excluded if they focused on non-porcine ECM sources, composite materials, reviews without primary data, or abstracts lacking full articles—the selection process involved screening titles and abstracts, followed by full-text reviews conducted by two independent reviewers. Disagreements were resolved through consensus or consultation with a third reviewer. Data extracted from the included studies encompassed publication year, ECM composition (e.g., collagen, GAGs, and growth factors), decellularization techniques (chemical, enzymatic, and physical), mechanical testing, immunological assays, and intended applications (e.g., wound healing, cardiovascular, and urologic).

In addition to general study characteristics, specific data relating to scaffold biochemical composition and mechanical properties were systematically extracted. Key biochemical markers included the presence and relative abundance of type I and III collagen, elastin fibers, glycosaminoglycan (GAG) concentrations, and growth factors such as vascular endothelial growth factor (VEGF) and basic fibroblast growth factor (b-FGF), when available. Information on decellularization methodologies, including enzymatic treatments, detergent-based protocols, and mechanical processing techniques, was also recorded. Where reported, mechanical testing outcomes such as tensile strength, elasticity, and compliance were documented. Studies were further categorized based on the targeted application domain, such as cardiovascular repair, urologic reconstruction, wound healing, or gastrointestinal surgery, to facilitate domain-specific synthesis and comparison.

Preclinical animal studies were evaluated using the SYRCLE risk of bias tool to assess quality and risk of bias, while clinical studies were appraised using the Joanna Briggs Institute (JBI) checklist [18,19]. Due to methodological heterogeneity across studies, data synthesis was conducted narratively. Descriptive statistics and thematic analysis were used to highlight emerging trends and inter-study variations.

Due to the substantial heterogeneity in study design, endpoints, and reporting formats, no quantitative meta-analysis or statistical hypothesis testing was conducted. Instead, descriptive statistics and thematic synthesis were employed to identify patterns in decellularization methods, validation strategies, and application domains. This qualitative approach was chosen to preserve fidelity to the data and to avoid misleading inferences from non-comparable outcomes.

## 5. Conclusions

The porcine small intestinal submucosa extracellular matrix (SIS-ECM) continues to demonstrate strong potential as a biomaterial platform in regenerative medicine due to its favorable structural composition, bioactive properties, and broad range of applications [4,5,7,8]. This meta-analysis, which incorporates 205 rigorously selected studies, highlights consistent findings in the preservation of key ECM constituents, such as collagens, glycosaminoglycans, and growth factors, which are essential for promoting cell migration, angiogenesis, and tissue remodeling [4,5].

Significantly, the field has progressed from basic descriptive work to complex, application-specific research designs [6,9,10,11]. Recent studies increasingly utilize in vivo models and functional metrics to evaluate scaffold integration and therapeutic efficacy, reflecting a growing commitment to translational relevance [10]. Concurrently, decellularization techniques have evolved from aggressive chemical protocols to more nuanced, multi-step approaches that prioritize both cellular clearance and ECM preservation [15,16].

Despite these advancements, challenges persist. Variability in decellularization protocols, donor tissue characteristics, and reporting standards continue to affect the reproducibility and clinical performance of SIS-ECM scaffolds [12,20]. Establishing consensus-based benchmarks for scaffold preparation and reporting would directly support clinical decision-making and facilitate regulatory alignment. Furthermore, concerns regarding immunogenic residues and mechanical failure in high-stress environments underscore the importance of robust product validation and long-term clinical data [13,14].

Future research should focus on standardizing processing and evaluation protocols, expanding clinical trials, and investigating combination therapies that involve SIS-ECM and bioactive agents or cells [3,6]. With these steps, SIS-ECM can advance toward a more predictable, safe, and effective use across various regenerative applications. Bridging the gap between laboratory research and routine clinical practice will require stronger collaboration between biomaterials scientists, surgeons, and regulatory agencies.

Through strategic refinement and interdisciplinary commitment, SIS-ECM may help shape the next era of biologically informed tissue engineering.

Compared to synthetic or allogeneic scaffolds, SIS-ECM offers distinct advantages, including its natural composition, high biocompatibility, and integrated bioactive factors that promote angiogenesis and remodeling. However, limitations such as variable mechanical strength and donor-to-donor variability remain challenges for certain applications. Future developments may emphasize hybrid strategies that combine SIS with biodegradable polymers (e.g., PLGA, PCL) or bioactive ceramics to enhance mechanical strength and degradation control. Additionally, targeted biological, chemical, or physical modifications—such as crosslinking, growth factor loading, or surface functionalization—could further improve the performance of SIS-based scaffolds for specific applications.

In addition to scientific and technical considerations, the broader clinical adoption of SIS-ECM will depend on economic and manufacturing factors. Cost-effective processing, consistent sourcing of raw materials, and scalable production protocols are essential for commercial viability. Furthermore, regulatory approval requires standardized documentation of quality, sterility, and biocompatibility, which must be addressed systematically to ensure broad accessibility in clinical settings.

Looking ahead, SIS-ECM is set to evolve through integration with next-generation technologies such as 3D bioprinting, smart biomaterials, and patient-specific tissue modeling. These innovations may enable the design of customized scaffolds with adjustable mechanical and biological properties. As computational modeling, stem cell biology, and biofabrication converge, SIS-derived ECMs could serve as foundational platforms for complex, hybrid regenerative therapies.

## Figures and Tables

**Table 1:** Distribution of SIS-ECM applications by field.

Field	Number of Studies	Percentage
Wound healing/dermal	70	34%
Cardiovascular	45	22%
Urology	31	15%
Gastrointestinal	24	12%
Dental/Oral/Maxillofacial	16	8%
Other	19	9%

**Table 2:** Frequency of decellularization methods.

Method Type	Technique	Studies
Chemical (detergents)	SDS, Triton X-100	41
Enzymatic	Trypsin, DNase/RNase	29
Physical	Freeze–thaw, pressure	14
Hybrid (multi-step)	Enzyme + detergent combo	23
Not specified	—	98

## Data Availability

The data presented in this study are available on request from the corresponding author.

## References

[1] Hynes R.O. (2009). The extracellular matrix: Not just pretty fibrils. Science.

[2] Frantz C., Stewart K.M., Weaver V.M. (2010). The extracellular matrix at a glance. J. Cell Sci..

[3] Badylak S.F., Taylor D., Uygun K. (2011). Whole-organ tissue engineering: Decellularization and recellularization of three-dimensional matrix scaffolds. Annu. Rev. Biomed. Eng..

[4] Shi L., Ronfard V. (2013). Biochemical and biomechanical characterization of porcine small intestinal submucosa (SIS): A mini review. Int. J. Burns Trauma.

[5] Fujii M., Tanaka R. (2022). Porcine small intestinal submucosa alters the biochemical properties of wound healing: A narrative review. Biomedicines.

[6] Mosala Nezhad Z., Poncelet A., de Kerchove L., Gianello P., Fervaille C., El Khoury G. (2016). Small intestinal submucosa extracellular matrix (CorMatrix) in cardiovascular surgery: A systematic review. Interact. Cardiovasc. Thorac. Surg..

[7] Casarin M., Fortunato T.M., Imran S., Todesco M., Sandrin D., Borile G., Toniolo I., Marchesan M., Gerosa G., Bagno A. (2022). Porcine small intestinal submucosa (SIS) as a suitable scaffold for the creation of a tissue-engineered urinary conduit. Int. J. Mol. Sci..

[8] Simman R. (2023). Role of small intestinal submucosa extracellular matrix in advanced regenerative wound therapy. J. Wound Care.

[9] Xu Q., Shanti R.M., Zhang Q., Cannady S.B., O’Malley B.W., Le A.D. (2017). A gingiva-derived mesenchymal stem cell-laden porcine SIS extracellular matrix construct promotes myomucosal regeneration of the tongue. Tissue Eng. Part A.

[10] Nherera L.M., Romanelli M., Trueman P., Dini V. (2017). An overview of clinical and health economic evidence regarding porcine SIS ECM in the management of chronic wounds and burns. Ostomy Wound Manag..

[11] Palmosi T., Tolomeo A.M., Cirillo C., Sandrin D., Sciro M., Negrisolo S., Todesco M., Caicci F., Santoro M., Dal Lago E. (2022). Small intestinal submucosa-derived extracellular matrix as a heterotopic scaffold for cardiovascular applications. Front. Bioeng. Biotechnol..

[12] Cobb M.A., Badylak S.F., Janas W., Simmons-Byrd A., Boop F.A. (1999). Porcine small intestinal submucosa as a dural substitute. Surg. Neurol..

[13] Jia Z., Wang Z. (2025). Photo-crosslinking hydrogel based on SIS decellularized matrix/fish collagen/GelMA. Int. J. Mol. Sci..

[14] Estrada Mira S., García-Briega M.I., Gómez Ribelles J.L., Restrepo Munera L.M. (2023). Viscoelastic properties of acellular matrices from porcine SIS and bovine pericardium. Materials.

[15] Crapo P.M., Gilbert T.W., Badylak S.F. (2011). An overview of tissue and whole organ decellularization processes. Biomaterials.

[16] Keane T.J., Swinehart I.T., Badylak S.F. (2015). Methods of tissue decellularization used for preparation of biologic scaffolds and in vivo relevance. Methods.

[17] Page M.J., McKenzie J.E., Bossuyt P.M., Boutron I., Hoffmann T.C., Mulrow C.D., Shamseer L., Tetzlaff J.M., Akl E.A., Brennan S.E. (2021). The PRISMA 2020 statement: An updated guideline for reporting systematic reviews. BMJ.

[18] Hooijmans C.R., Rovers M.M., De Vries R.B., Leenaars M., Ritskes-Hoitinga M., Langendam M.W. (2014). SYRCLE’s risk of bias tool for animal studies. BMC Med. Res. Methodol..

[19] Moola S.Z., Munn Z., Tufanaru C., Aromataris E., Sears K., Sfetcu R., Currie M., Qureshi R., Mattis P., Lisy K.M., Aromataris E., Munn Z. (2020). Systematic reviews of etiology and risk. JBI Manual for Evidence Synthesis.

[20] Zheng M.H., Chen J., Kirilak Y., Willers C., Xu J., Wood D. (2005). Porcine small intestine submucosa (SIS) is not an acellular collagenous matrix and contains porcine DNA: Possible implications in human implantation. J. Biomed. Mater. Res. B Appl. Biomater..

